# A Rare Case of Concurrent Primary Biliary Cholangitis and Sarcoidosis

**DOI:** 10.7759/cureus.80228

**Published:** 2025-03-07

**Authors:** Robert S Gordon, Mrudula Bandaru, Laxmikausthubha Yaratha, Marie L Borum, Mamoun Younes

**Affiliations:** 1 Internal Medicine, George Washington University School of Medicine and Health Sciences, Washington, USA; 2 Gastroenterology and Liver Diseases, George Washington University School of Medicine and Health Sciences, Washington, USA; 3 Pathology, George Washington University School of Medicine and Health Sciences, Washington, USA

**Keywords:** case report, granulomatous liver disease, hepatic sarcoidosis, peripheral eosinophilia, primary biliary cholangitis (pbc)

## Abstract

Primary biliary cholangitis and sarcoidosis are the two most common causes of granulomatous liver disease in the US; however, their expected clinical course and management are starkly different. This case involves a patient with granulomas noted on liver biopsy, presumed secondary to known sarcoidosis, later found to have concurrent primary biliary cholangitis. Prompt diagnosis of primary biliary cholangitis is crucial as its natural course of progressive liver fibrosis can be significantly improved with treatment.

## Introduction

Granulomatous liver disease is characterized by the presence of focal aggregates of macrophages within the liver [1]. The most common causes of granulomatous liver disease within the United States include sarcoidosis, primary biliary cholangitis (PBC), mycobacterial infection, and drug-induced liver injury [1]. While certain characteristics of granulomas seen on histology may provide hints toward the etiology, it is not sufficient for diagnosis. Determination of the etiology of granulomatous liver disease is dependent upon clinical presentation, patient history, and other diagnostic evaluations. 

Sarcoidosis is a multisystem non-caseating granulomatous disease in which 90% of patients exhibit pulmonary findings such as hilar adenopathy [2]. Diagnosis of sarcoidosis is determined by three criteria: clinical presentation, presence of non-caseating granulomas on biopsy, and exclusion of other granulomatous diseases [2]. Sarcoidosis can be diagnosed in the absence of biopsy in the case of pathognomonic clinical presentations such as Löfgren's syndrome, lupus pernio, or Heerfordt’s syndrome [2]. Heerfordt’s syndrome is a rare condition characterized by facial palsy, parotid gland enlargement, and uveitis, and is highly specific for sarcoidosis [2]. Hepatic involvement in sarcoidosis is seen in roughly 3.6%-20% of patients, typically presenting as asymptomatic elevations in alkaline phosphatase and liver enzymes [3]. However, post-mortem studies have demonstrated the presence of granulomatous liver disease in upward of 80% of patients with sarcoidosis, suggesting a high prevalence of non-clinically significant liver involvement in patients with sarcoidosis [3]. The occurrence of significant impairment of liver function secondary to hepatic sarcoidosis is relatively rare with glucocorticoids and other immunomodulatory therapies as the mainstay of treatment [3].

PBC is a cholestatic liver disease caused by T-cell-mediated destruction of interlobular biliary ducts with a high risk of progression to cirrhosis if left untreated [4]. PBC is typically asymptomatic at time of diagnosis, often identified due to elevations in liver function labs, most notably alkaline phosphatase [4]. If present, symptoms are consistent with cholestasis, including fatigue, pruritis, abdominal pain, and jaundice. Diagnosis is often achieved without the need for biopsy as its serologic hallmark, the anti-mitochondrial antibody (AMA), is positive in 90%-95% of cases [4]. In the absence of AMA positivity, PBC specific antinuclear antibodies (ANAs) anti-sp100 and anti-gp210 or a biopsy displaying non-suppurative destructive cholangitis can be diagnostic [4]. The mainstay of treatment is ursodeoxycholic acid which results in significant improvement in transplant-free survival rates [4]. The coexistence of PBC and sarcoidosis is a relatively rare occurrence; however, several cases have been documented in the past suggesting a potential common pathway for the two granuloma-forming diseases with poorly understood etiologies [5-7].

## Case presentation

A 62-year-old female with a history of sleeve gastrectomy and sarcoidosis diagnosed in 2007 in the setting of Heerfordt syndrome, later confirmed via skin biopsy, was found to have an elevated alkaline phosphatase and transaminitis. At time of presentation, the patient was not taking any glucocorticoids or other immunomodulatory medications. The patient was asymptomatic and denied abdominal pain, nausea, vomiting, pruritus, and fatigue. The patient denied any knowledge of prior history of liver disease. Abdominal ultrasonography was performed and did not demonstrate any intrahepatic or extrahepatic ductal dilation.

**Figure 1:**
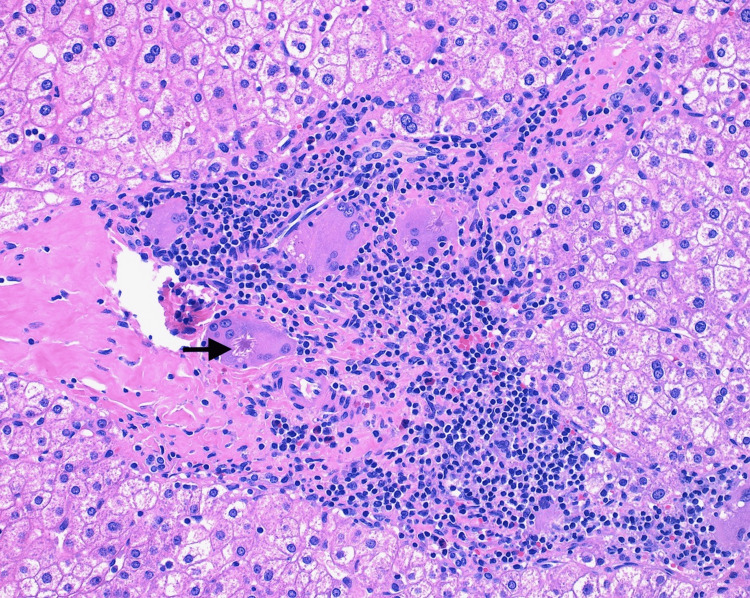
Portal tract with granulomatous inflammation. One of the multinucleate giant cells shows an asteroid body (black arrow). H&E stain, 20x microscope objective.

One year prior, the patient underwent gastric sleeve placement for obesity management with a liver biopsy collected at that time for reported “chronic liver disease,” which was not documented elsewhere. The pathology report documented that sections of the liver biopsy showed several multinucleated giant cells scattered mostly around portal tracts with occasional aggregates of giant cells present. Some of the giant cells contained intracytoplasmic asteroid bodies (Figure 1). Pedal microgranulomas were also present within the hepatic parenchyma. There was also patchy infiltration of and expansion of portal tracts by mixed chronic inflammatory cell infiltrate with occasionally prominent numbers of plasma cells and eosinophils without significant interface hepatitis, and mild patchy lobular inflammation. Occasional bile ducts demonstrated infiltration by neutrophils. There were 5%-10% macrovesicular steatosis without ballooned hepatocytes or Mallory bodies. Occasional bile duct hamartomas (von Meyenburg complex) were also present. There was no cholestasis, ductopenia, atypia or malignancy noted. The iron stain was negative. PAS stain with diastase was unremarkable; alpha-1 antitrypsin globules were not present. The reticulin stain was unremarkable. The trichrome stain demonstrated no significant increase in hepatic fibrosis. These changes were thought to be suspicious for infiltrative sarcoidosis at the time due to the patient’s history.

The patient's alkaline phosphatase was found to be 1,076 IU/L, gamma-glutamyltransferase (GGT) 591 IU/L, aspartate aminotransferase (AST) 124 IU/L, and alanine aminotransferase (ALT) 196 IU/L with prior normal liver function testing eight months prior (Table 1). Other notable laboratory findings include an erythrocyte sedimentation rate (ESR) of 130 mm/hr, C-reactive protein of 40.8 mg/L, and peripheral eosinophilia with 35% eosinophils noted with an eosinophil count of 3.71 x 10^3^/mcL on manual read (Table 1). The patient’s current laboratory findings are consistent with cholestatic liver injury with peripheral eosinophilia which prompted testing for etiologies other than sarcoidosis such as auto-immune hepatitis, IgG-4-related disease, and PBC. The patient was found to have a positive AMA consistent with PBC. The patient was scheduled to follow closely with a local hepatology clinic for continued observation and management where she was initiated on ursodeoxycholic acid therapy.

**Table 1:** Significant laboratory findings.

Test	Patient values	Reference range
Alkaline phosphatase	1,076 IU/L	40-125 IU/L
Gamma-glutamyltransferase	591 IU/L	10-45 IU/L
Aspartate aminotransferase	124 IU/L	15-50 IU/L
Alanine aminotransferase	126 IU/L	10-45 IU/L
Erythrocyte sedimentation rate	130 mm/hr	0-30 mm/hr
C-reactive protein	40.8 mg/L	0-9 mg/L
Eosinophil # manual	3.71 x 10^3^/mcL	0.0-0.65 x 10^3^/mcL
Eosinophil % manual	35%	1%-5%

## Discussion

This case demonstrates the need to retain a broad differential in evaluation of granulomatous liver disease, even in the setting of a known cause of granulomatous liver disease such as sarcoidosis. While sarcoidosis may involve any organ system, clinically significant liver disease secondary to sarcoidosis is rare with very limited studies into management [3]. The mainstay of treatment for hepatic sarcoidosis is glucocorticoids and other immunomodulatory medications such as methotrexate [3].

In contrast to the relatively mild impact of sarcoidosis on the liver, PBC causes the progressive destruction of interlobular biliary ducts, resulting in significant liver disease [4]. Without treatment patients progress by one histological stage every 1.5 years on average with 50% of patients identified at an early stage developing cirrhosis within four years [8]. Ursodeoxycholic acid (UDCA) is the first-line therapy for PBC. One study demonstrated significant improvement in five-, 10-, and 15-year liver transplant-free survival rates with 90%, 78%, and 66% survival with UDCA treatment vs. 79%, 59%, and 32% survival without treatment, respectively [9]. Management with UDCA has also demonstrated symptom improvement, improved liver biochemistries, slowed progression of liver fibrosis, and delayed onset of esophageal varices [9-11]. Early detection and management of PBC is critical to improve prognosis. While nonspecific, peripheral eosinophilia is a common finding in PBC and can be a useful clue in the early stages of the disease, as was the case with this patient [12]. Eosinophilic liver infiltration has a variety of potential etiologies such as parasitic infections and drug hypersensitivities. However, several studies have noted an association between PBC and eosinophilic infiltration into portal tracts as seen in this case [13-15]. 

While rare, the coexistence of PBC and hepatic sarcoidosis has been noted in several case reports [5-7,16]. The shared features of intrahepatic granuloma formation and cholestatic liver injury present a diagnostic challenge in these cases. Given the progressive nature of PBC and the availability of an effective treatment, PBC must be ruled out in the setting of hepatic granulomas seen on biopsy, even in the setting of known sarcoidosis. The pathogenesis of both sarcoidosis and PBC is poorly understood and further investigation into the cooccurrence and clinical overlap of these two disease processes may shed light on a common pathway. Prior case reports have suggested the potential of a shared defect in cell-mediated immunity prompting granuloma formation [16].

## Conclusions

This case highlights the need for maintaining a broadened differential diagnosis and a full evaluation of granulomatous liver disease, despite the presence of a known granulomatous disease. This case also demonstrates that peripheral eosinophilia and eosinophilic infiltration of portal tracks can be useful hints towards diagnosing PBC. The prompt identification of PBC is crucial as the resulting progressive liver damage can be significantly delayed with timely initiation of proper therapy.
